# Synchrony of Clinical and Laboratory Surveillance for Influenza in Hong Kong

**DOI:** 10.1371/journal.pone.0001399

**Published:** 2008-01-02

**Authors:** Lin Yang, Chit Ming Wong, Eric H. Y. Lau, King Pan Chan, Chun Quan Ou, Joseph S. M. Peiris

**Affiliations:** 1 Department of Community Medicine, The University of Hong Kong, Hong Kong Special Administrative Region, China; 2 Department of Microbiology, The University of Hong Kong, Hong Kong Special Administrative Region, China; University of Cape Town, South Africa

## Abstract

**Background:**

Consultation rates of influenza-like illness (ILI) in an outpatient setting have been regarded as a good indicator of influenza virus activity in the community. As ILI-like symptoms may be caused by etiologies other than influenza, and influenza virus activity in the tropics and subtropics is less predictable than in temperate regions, the correlation between of ILI and influenza virus activity in tropical and subtropical regions is less well defined.

**Methodology and Principal Findings:**

In this study, we used wavelet analysis to investigate the relationship between seasonality of influenza virus activity and consultation rates of ILI reported separately by General Out-patient Clinics (GOPC) and General Practitioners (GP). During the periods 1998–2000 and 2002–2003, influenza virus activity exhibited both annual and semiannual cycles, with one peak in the winter and another in late spring or early summer. But during 2001 and 2004–2006, only annual cycles could be clearly identified. ILI consultation rates in both GOPC and GP settings share a similar non-stationary seasonal pattern. We found high coherence between ILI in GOPC and influenza virus activity for the annual cycle, but this was only significant (*p*<0.05) during the periods 1998–1999 and 2002–2006. For the semiannual cycle high coherence (*p*<0.05) was also found significant during the period 1998–1999 and year 2003 when two peaks of influenza were evident. Similarly, ILI in GP setting is also associated with influenza virus activity for both the annual and semiannual cycles. On average, oscillation of ILI in GP and of ILI in GOPC preceded influenza virus isolation by approximately four and two weeks, respectively.

**Conclusions:**

Our findings suggest that consultation rates of ILI precede the oscillations of laboratory surveillance by at least two weeks and can be used as a predictor for influenza epidemics in Hong Kong. The validity of our model for other tropical regions needs to be explored.

## Introduction

Influenza has been associated with a heavy burden on morbidity and mortality, both in the temperate and subtropical/tropical regions [1]–[4]. Surveillance of influenza disease burden has been identified to be critically important by the World Health Organization (WHO) Global Agenda on Influenza [5]. However, non-specific symptoms of influenza infection reduce the reliability for early detection of influenza epidemics based on increased consultation rates of influenza-like illness (ILI). It has been reported that none of the ILI symptoms, except fever, could reliably differentiate influenza infections from those caused by other etiologies [6]. Selected combinations of the ILI symptoms could provide better predictions, for example, fever and cough together have been shown to correctly predict at least 79% of influenza [7]. A study in UK found only about 30% of specimens from patients presenting with ILI symptoms in a general practitioner's surveillance network were positive for influenza [8]. Laboratory surveillance, which is believed to provide more accurate information about influenza virus activity than clinical surveillance, has been implemented in parallel with clinical surveillance in many regions [9], [10]. Unfortunately, due to constraints of time and budget, laboratory surveillance tends to be more restricted in scale with relatively smaller numbers of patients being tested with the primary aim of detecting genetic and antigenic changes of circulating strains in order to update influenza vaccines rather than for early warning of influenza epidemics [11]. Furthermore, in many parts of the world, there is no laboratory surveillance at all, thus emphasizing the need for validated alternatives.

Previous studies about the predictability of influenza activity from clinical surveillance of ILI were mainly focused on the sensitivity and specificity of case definition for ILI to laboratory-confirmed influenza infection [7], [12]–[14]. For the purpose of predicting when influenza epidemics occur, it is probably of more interest to examine whether increase of ILI consultation rates in clinics precedes the increase of influenza virus activity in the community. In most temperate regions, where influenza epidemics occur in relatively fixed periods (mostly winter time), the prediction of influenza epidemics based on ILI consultation rates is reliable as ILI data were well correlated with laboratory data [15]. However, in the tropics and subtropics, both ILI rates and influenza virology surveillance data exhibit non-stationary seasonality, with unpredictable peaks appearing during winter to summer [16]. As a result, a better strategy is required to test the association between clinical and laboratory surveillance data in these regions. Contemporary statistical techniques such as wavelet analysis may be usefully applied to this problem. Wavelet analysis has been first adopted into the fields of climatology [17], [18] and has only recently been introduced into the ecology of infectious diseases such as measles [19], pertussis [20], dengue fever [21] and influenza [22]. By decomposing a time series into the various time-frequency spaces, wavelet analysis is more suitable for modeling nonstationary seasonality of a time series than traditional Fourier analysis; therefore in this study we used wavelet analysis to model the seasonal variation of ILI rates and virology surveillance data.

In this study, we took advantage of the long-standing sentinel surveillance system in Hong Kong, to quantify the synchrony between ILI consultation rates and the virus activity revealed by laboratory surveillance. Hong Kong is located at the 22°18′N and 114°10′E and has a typical subtropical climate with four well-separated seasons. Hong Kong has a well defined population of 6.9 million in a total area of 1,095 sq. km, which can be regarded as relatively spatio-temporally homogenous for a rapidly spread infection such as influenza. Moreover, being geographically close to southern China, Hong Kong is regarded as a sentinel post adjacent to the hypothetical influenza epicenter for the emergence of pandemic influenza [23]. Therefore, surveillance for influenza in Hong Kong is critical to global influenza pandemic preparedness. We used wavelet analysis to compare the timing and magnitude of oscillations of these time series, and showed that ILI consultation rates was in synchrony with laboratory surveillance and therefore can be adopted as a reliable indicator for influenza epidemics.

## Methods

### Data

In 1998 the Department of Health (DH) in Hong Kong enhanced its sentinel surveillance program for influenza [24]. Influenza-like illness was defined as symptoms of fever ≥38°C, cough and/or sore throat, similar to the definition adopted by the Centers for Disease Control and Prevention (CDC) of the United States [25]. Each week the consultation rates of patients with ILI symptoms were reported by General Out-patient Clinics (GOPC, 64 reporting clinics since 1998) and General Practitioners (GP, 18–30 reporting clinics in 1998–1999 and 50 since 2000) selected throughout Hong Kong. About 57% of total outpatient consultations in Hong Kong are provided by GP and about 24% by GOPC [26]. Laboratory surveillance was implemented at the same time, in which respiratory specimens were collected from ILI patients in GP and GOPC settings as well as patients hospitalized with acute respiratory diseases. Those hospitalized patients were all from the publicly funded hospitals, which cover 95% of bed-days in Hong Kong [27]. The total number of specimens and number of specimens positive for influenza virus A (subtypes H1N1 and H3N2) and B were recorded according to their sampling dates [28]. An average of 21,000 specimens (ranging from 12,000 to 41,000) was tested annually in laboratory surveillance. We downloaded the weekly ILI consultation rates in both GOPC and GP from January 1998 to May 2006 from the open-access website of DH [24] and obtained laboratory surveillance data during the same period from the Government Virus Unit, DH.

### Wavelet analysis

Wavelet analysis allows decomposition of a time series into the time-frequency space and therefore is better able to detect the variation of periodicity over time [29]. Statistical significance can be determined by comparing the power of the decomposed time series with red-noise over different frequency and time. Similar to Fourier coherence, wavelet coherence examines the association of two time series in time and frequency, i.e., whether two time series oscillate simultaneously, but without the assumption of stationarity such that temporal changes in the association can also be estimated. High coherence suggests the capability of one time series to predict the other one [30]. In wavelet coherence analysis, wavelet transforms have to be normalized to ensure that they are comparable to each other [29]. By calculating the phase difference from wavelets cross the whole spectrum, we were able to quantify the lag period between two time series [30]. Wavelet analysis and wavelet coherence were conducted in R using the sowas and Rwave packages [31]–[33]. The detailed theory for wavelet analysis has been described by Torrence *et al.* [29]

### Ethics

The ethical approval has been obtained from the Institutional Review Board of the University of Hong Kong/Hospital Authority Hong Kong West Cluster (Reference No. UW 04-250 T/572).

## Results

**Figure 1:**
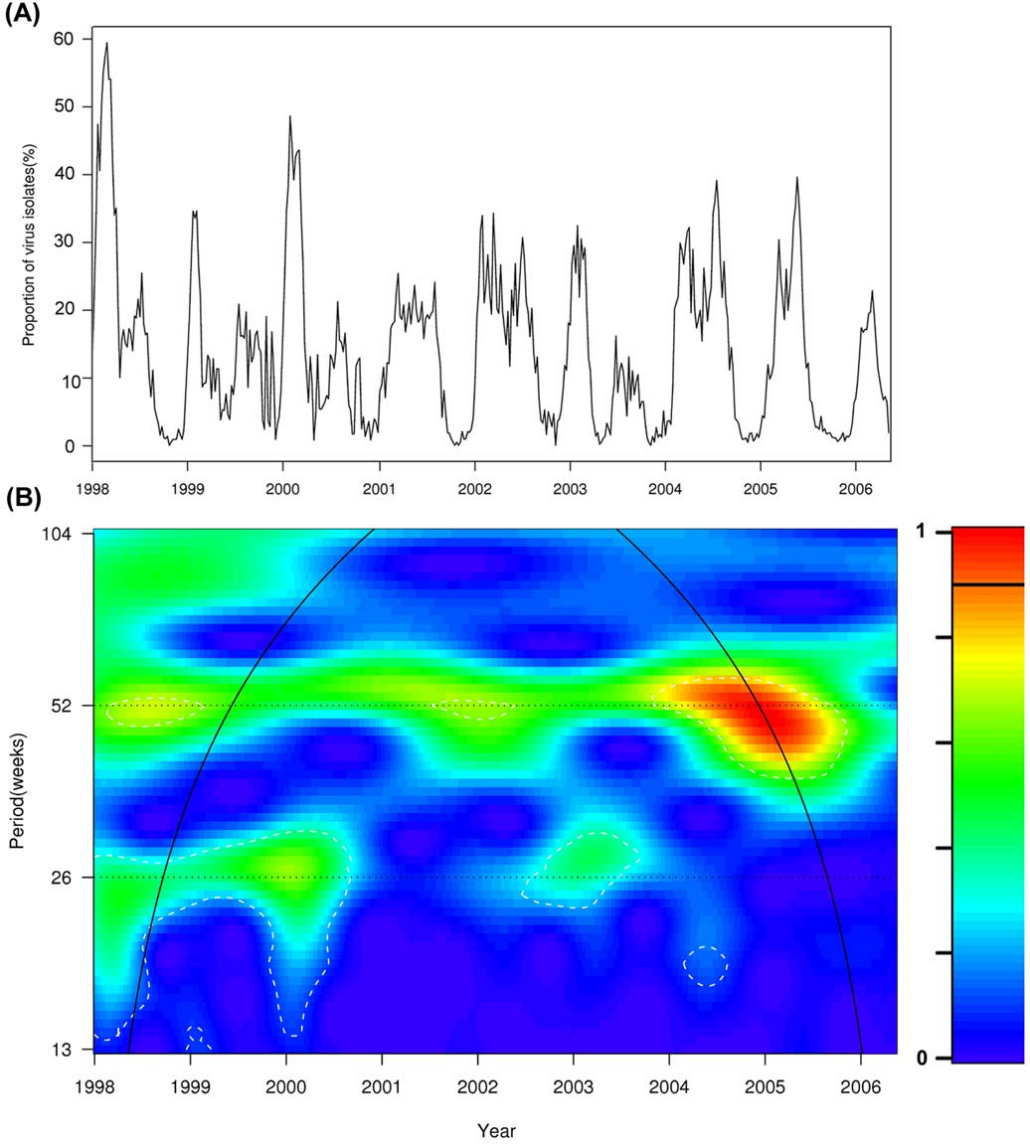
Wavelet analysis for the weekly proportions of specimens positive for influenza. (A)The time series of weekly proportions; (B) Wavelet power spectrum. The white broken-line contour lines show the regions of power significant at the 5% level computed based on 1,000 Monte Carlo simulations. The cone of influence (black curve) indicated the region without edge effects. The power values were coded from dark blue for low power to dark red for high power, as shown in the right panel.

Influenza activity, which was measured by the weekly proportions of specimens positive for influenza compiled from laboratory surveillance data, is showed in Figure 1A. The mean of influenza isolation proportions was 13.8% and ranged from 0% to 59.3%. The means for ILI consultation rates in GOPC and GP were 5.6 (range: 1.0–19.7) and 47.5 (range: 22.9–123.0) per 1,000 consultations. The monthly averages of influenza isolation proportions and ILI consultation rates were summarized in Table 1. As shown in wavelet analysis (Figure 1B), in the years of 1998–2000 and 2003, influenza activity showed a statistically significant semiannual pattern (*p*<0.05), with one peak in winter and another in late spring or early summer; while in the years of 2001, 2002 and 2004–2006, only an annual seasonal pattern was noticeable. ILI consultation rates in both GOPC and GP showed seasonal patterns similar to influenza virus activity (Figure S1 and S2).

**Table 1:** Monthly averages of influenza isolation proportions and ILI consultation rates in GOPC and GP settings, 1998–2006.

Month	Proportions of Influenza (%)	ILI consultation rates (per 1,000)	
		GOPC	GP
January	17.9	6.1	52.8
February	27.4	8.0	58.7
March	25.3	7.8	58.2
April	14.7	5.0	47.5
May	14.1	5.9	49.6
June	16.3	6.5	48.4
July	18.2	6.4	45.8
August	12.9	4.8	41.4
September	6.5	4.4	43.2
October	2.2	4.0	41.6
November	1.5	3.9	41.4
December	3.4	4.1	42.9

**Figure 2:**
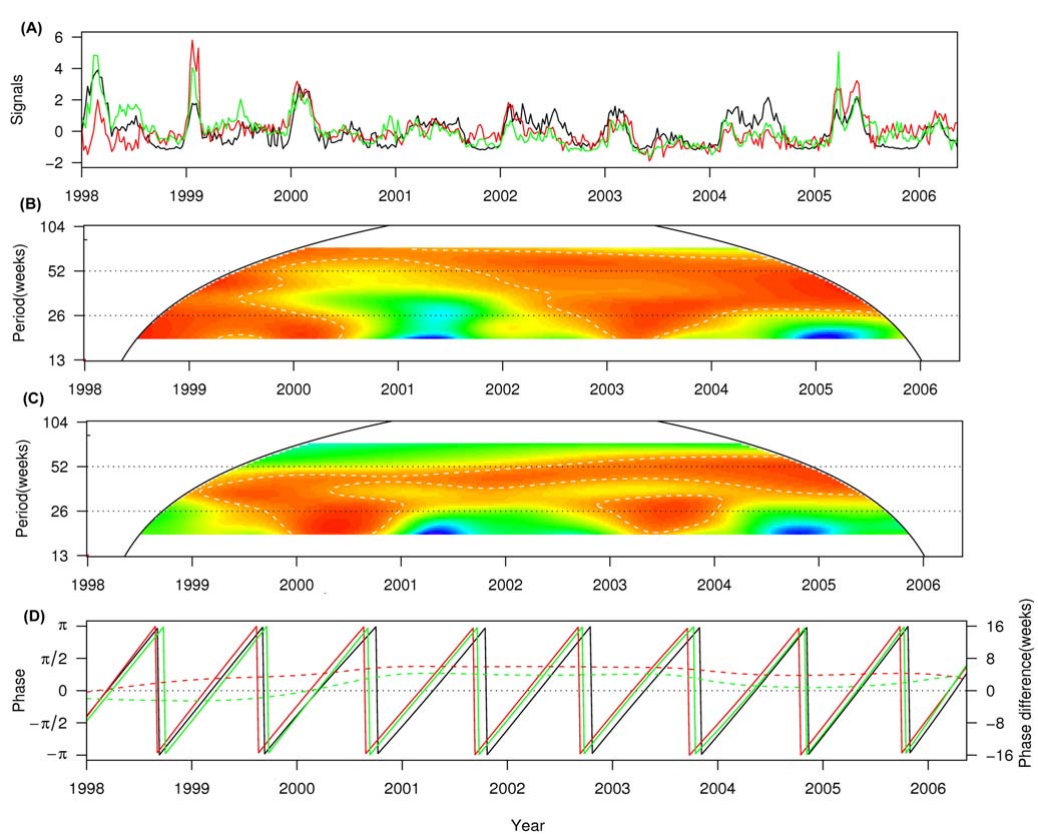
Association between ILI consultation rates (per 1,000 consultations) and influenza virus isolation. (A) The normalized ILI consultation rates in GOPC (green) and in GP (red), and influenza virus isolation (black); (B) Wavelet coherence between ILI in GOPC and influenza virus isolation. The power values were colored as Figure 1(B). The white broken-line contour lines show the regions of power significant at the 5% level computed based on 1,000 Monte Carlo simulations. The cone of influence (black curve) indicated the region without edge effects. (C) Wavelet coherence between ILI in GP and influenza virus isolation (colors as in (B)). (D) Phases of time series (solid lines, colors as in (A)) at the annual cycle (48–56 week band) and phase difference between ILI in GOPC and influenza virus isolation (green dashed line), between ILI in GP and influenza virus isolation (red dashed line).

Results of wavelet coherence between ILI in GOPC or ILI in GP and influenza activity are shown in Figure 2. ILI consultation rates in GOPC presented a consistently significant coherence with influenza activity at the annual cycle (*p*<0.05) throughout the study period. For the semiannual cycle, high coherence (*p*<0.05) was found in the years 1998, 1999 and 2003 when annual seasonality of influenza was evident (Figure 2B). A similar pattern of coherence was also observed between ILI consultation rates in GP and virus activity at both the annual and semiannual cycles (Figure 2C).

To compare the timing for oscillation of ILI in GOPC and ILI in GP to influenza activity, we calculated their phase difference at the annual cycle (52-week periodic band), which was the only cycle consistently evident throughout the whole study period. In general, ILI consultation rates in both GOPC and GP were in synchrony with influenza activity. Influenza activity was estimated to follow ILI in GOPC with an average delay of 1.6 week (ranged from −2.4 to 4.2 weeks) and to follow ILI in GP with an average delay of 4.1 weeks (ranged from −0.3 to 6.0 weeks). Since the annual seasonality of influenza activity did not always cycle exactly at a 52-week frequency, we also calculated the phase difference at the 48–56-week periodic band. The estimated phase differences had averages quite close to those at the 52-week periodic band (1.6 weeks for GOPC and 4.3 weeks for GP), but with slightly larger ranges (−2.5 to 4.3 weeks for GOPC and −0.3 to 6.0 weeks for GP) (Figure 2D). Oscillation of ILI in GOPC lagged by 2.3 weeks behind that of influenza activity at the beginning of study period (1998), surpassed influenza activity in the year 2000, and stabilized to around 3 weeks before influenza activity after 2000. We did not find any obvious turning point for the phase difference between ILI in GP and influenza activity. ILI in GP consistently preceded influenza activity by 4–5 weeks except approaching the latter at the beginning (1998) and end (2006) of the study period.

## Discussion

As in most subtropical/tropical regions, influenza circulation in Hong Kong presents a less well-defined seasonality when compared with the temperate countries [16]. In the years 1998–2000 and 2003, influenza virus activity detected through laboratory surveillance clearly demonstrated both annual and semiannual cycles, but in the periods 2001–2002 and 2004–2006, only an annual cycle was evident. By using Fourier decomposition, Alonso and colleagues were able to detect a major annual cycle and minor semiannual cycle in the seasonality of pneumonia and influenza mortality in Brazil [34]. However, they did not observe any transition of seasonal patterns. The major difference between years with annual or semiannual peaks was not the overall period of influenza virus activity, but whether this period continued as one broad peak or was decomposed into two distinct periods of viral circulations. However, even in these years of semiannual peaks, the dominant virus in each peak was the same subtype A(H3N2) (Figure S3). It is interesting that the emergence of a semiannual cycle in Hong Kong coincided with significant antigenic change in influenza A(H3N2) viruses. The 1997–1998 season was marked by a significant antigenic drift, with the transition from A/Wuhan/359/95-like viruses to the A/Sydney/5/97-like viruses. The latter was dominant until 2000. Another significant antigenic change, from the A/Sydney/5/97-like viruses to the A/Fujian/411/2002-like viruses, occurred in the 2002–2003 season when the semiannual seasonality became evident again [35]. The semiannual peaks of virus activity were better defined with sharp spikes whereas the annual peaks tended to be broad and shallow. Therefore, it is likely that the semiannual cycle was caused by a lack of herd immunity when new virulent H3N2 strains were introduced into human population. However, our study only covered the period from 1998 to 2006 most of which were dominated by H3N2 viruses, therefore, we could not rule out the possibility that H1N1 may also account, at least partially, for the variation of influenza seasonality. Further studies in other subtropical/tropical regions may shed more light on mechanisms of transition of influenza seasonality.

The primary aim of sentinel surveillance is to provide timely alerts for outbreaks of influenza infections. A recent study in Hong Kong compared different short-term models in terms of their sensitivity, specificity and timeliness to detecting influenza outbreaks based on the same clinical surveillance data as we used in this study [28]. The authors found the models based on short-term clinical surveillance data from GP could provide specific and sensitive alerts within the two weeks of peak seasons confirmed by laboratory surveillance data. In line with their results, we found high synchrony between laboratory and clinical surveillance, in both GP and GOPC. Therefore, consultation rates of ILI could be used a reliable and timely indicator for virus activity in terms of both long-term and short-term variations. Their investigations focused their analysis within a short-time period when virus activity exceeds a pre-determined threshold. However, owing to lack of well-defined seasonality of influenza in the tropics and subtropics, it can be difficult to define an epidemic threshold based on historical data [4], [28]. In this study we analyzed the data covering longer than eight years of the time series, and avoided arbitrarily defining the epidemic periods. We estimated that laboratory surveillance lagged by approximately 4 weeks following increase of ILI consultation rates in GP, and by approximately 2 weeks following increase of ILI rates in GOPC. The phase difference between clinical and laboratory surveillance was rather consistent for ILI in GP, but not for ILI in GOPC. The change in phase difference could perhaps be partially explained by the edge effects, which are common in the time series analysis because of the lack of data beyond both ends. Moreover, due to a large number of outpatient visits in GOPC for chronic problems or following up visits, the ILI consultation rates were likely under-represented and thereby less timely than the rates reported by GP.

Synchrony between ILI consultation rates and influenza virus activity may potentially be affected by the seasonality of other respiratory viruses which may also cause symptoms of ILI. Among these viruses, respiratory syncytial viruses (RSV) are probably of the greatest concern, especially in regard to children. RSV has been found to co-circulate with influenza viruses in the temperate regions, and in some years RSV even accounted for more ILI cases than influenza in the young children [8]. However, in Hong Kong, RSV tends to be active in the spring and summer, and peaks of RSV usually occur weeks after those of influenza (Figure S4). Given its seasonality, RSV may potentially prolong the peaks of ILI in both GOPC and GP, and thereby reduce the synchrony between ILI and influenza virus activity. Virological diagnosis for RSV is not done in the ILI surveillance program but is available for hospitalized patients. We conducted a similar wavelet analysis using the combined RSV and influenza data from hospitalized patients and found that the combined influenza and RSV virus activity was out of phase with ILI rates in both GOPC and GP settings. RSV alone was also out of phase with ILI datasets (data not shown). It is possible that RSV contributed little to the ILI cases in outpatients because infants and young children do not contribute significantly to this data set. Since the Hong Kong Department of Health does not collect the age information of ILI patients included in the clinical surveillance, we have no means of verifying this hypothesis.

Accounting for the lag from infection to admission, our results show that upsurges in ILI consultation rates in GP preceded increase in influenza virus isolation rates detected by laboratory surveillance by at least 3 weeks consistently. The laboratory surveillance was dominated by specimens submitted from patients hospitalized with acute respiratory infections, therefore reflecting more severe complications of influenza. Thus the 3 week lag between ILI in GP and laboratory surveillance may simply reflect a threshold effect where mild infections are detected early in the outpatient surveillance system, while the upsurge in hospitalized cases needs the build-up of sufficient numbers of cases before the more severe complications of disease would trigger hospital admissions. The alternative hypothesis which cannot be excluded is that the severity of influenza increases with the buildup of the epidemic. The sequence of oscillation waves, transmitting from GP to GOPC then, is not surprising. Patients in Hong Kong tend to visit GP immediately after onset of respiratory symptoms. If the symptoms are severe or deteriorating, they may switch to GOPC where later they could probably be admitted into hospitals.

Our findings provide insight into methods used for influenza surveillance. Clinical surveillance in outpatients could be used as a good indicator for influenza virus activity. More specifically, we expect clinical surveillance in GP outperforms GOPC in terms of providing a timelier alert for precautionary measures. In future, a prediction model incorporating the mildly changing coherence between clinical and laboratory surveillance may be developed on the basis of wavelet analysis to promptly forecast the influenza outbreaks.

We need to be cautious while interpreting the virus activity in 2003 when the health care seeking pattern of Hong Kong residents was influenced by the SARS outbreak. In fact, an average of 600 specimens were collected within one week for laboratory surveillance in the first half year of 2003, two times as high as the previous average weekly level. The weekly numbers of specimens decreased to around 200 per week in the second half year of 2003. Such a dramatic change in sampling numbers may bias the influenza seasonality estimated according to influenza isolation rates from laboratory surveillance, and also may likely bias the ILI consultation rates in outpatients. Despite the potential bias associated with the data in 2003, we are still confident of the results of wavelet analysis for the other years of our study period. Because in wavelet analysis the seasonal patterns of the time series were separately examined in numerous fixed time-frequency windows, the estimate from one window should not interfere with those from the neighboring windows. Besides, in our study, rates and proportions were used which may not be affected by the variations in sample sizes in the years concerned.

In conclusion, we demonstrated that ILI consultation rates in both GOPC and GP oscillated consistently ahead of laboratory surveillance, which in our dataset, predominantly cases from hospitalized patients. Therefore, clinical sentinel surveillance could be a reliable and early indicator of changes in influenza virus activity. Wavelet analysis revealed changes of influenza seasonality patterns when a novel antigenic virus variant emerged. This approach could be applied to the temperate and other tropical regions, to provide more insight into the mechanisms of seasonal appearance of influenza viruses.

## Supporting Information

Figure S1 Wavelet analysis for the weekly consultation rates of influenza-like illness in GOPC.
(7.38 MB TIF)

Figure S2 Wavelet analysis for the weekly consultation rates of influenza-like illness in GP.
(6.94 MB TIF)

Figure S3 Weekly proportions of isolates positive for influenza subtypes: A(H3N2) (red), A(H1N1) (green), B(blue) and overall proportions (black), 1998-2006. Data were collected from the Department of Health.
(3.60 MB TIF)

Figure S4 Weekly proportions of isolates positive for influenza (black) and RSV (red), 1998-2003. Data were collected from the Queen Mary Hospital, one of major public hospitals in sentinel surveillance systems of Hong Kong.
(3.80 MB TIF)
