# Supplementary figures and images for: Synchrony of Clinical and Laboratory Surveillance for Influenza in Hong Kong
Source: PLoS One. 2008 Jan 2;3(1):e1399. doi: 10.1371/journal.pone.0001399 (PMC2151138; doi:10.1371/journal.pone.0001399)

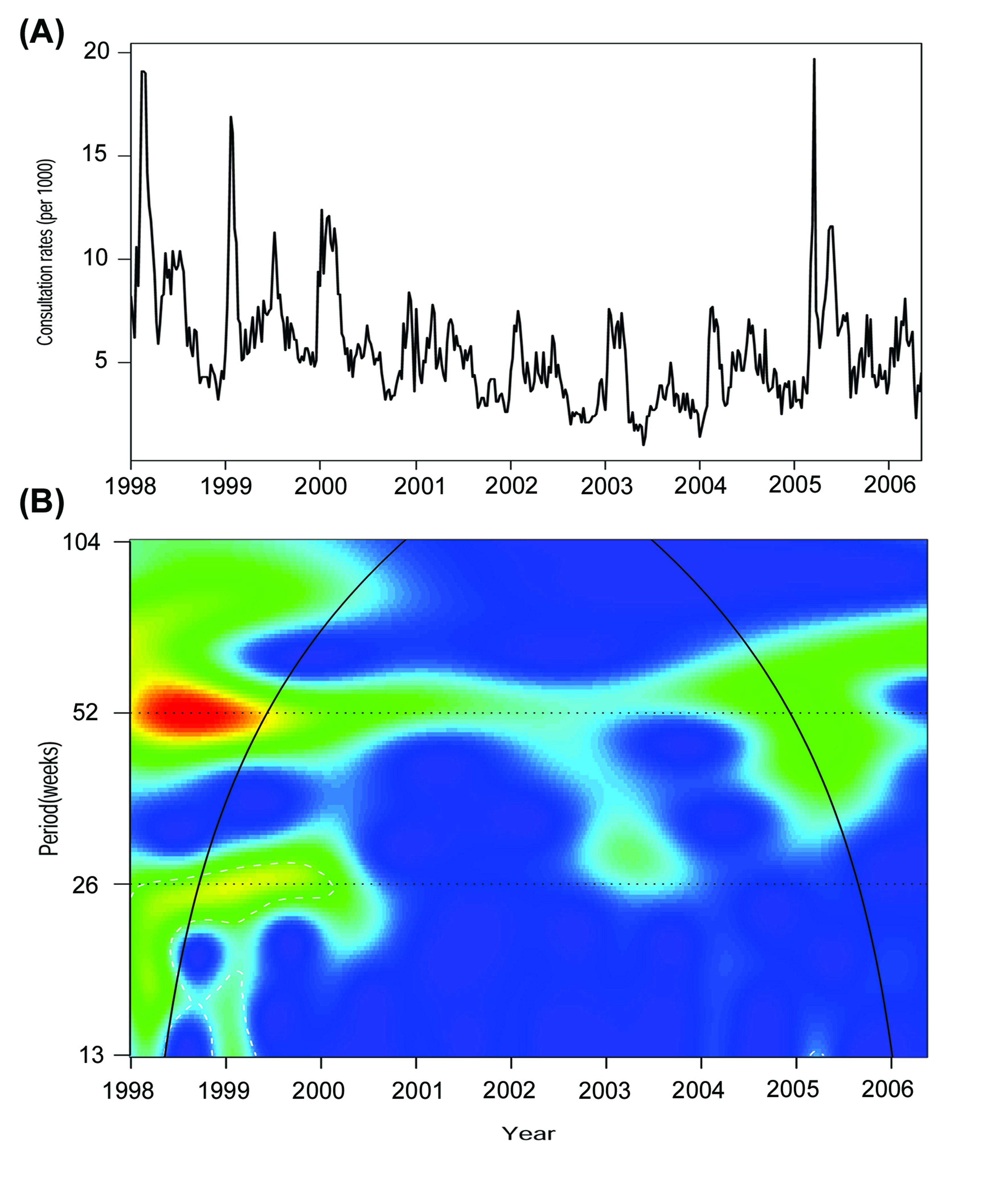

Supplement: Figure S1 — Wavelet analysis for the weekly consultation rates of influenza-like illness in GOPC. (7.38 MB TIF) [file pone.0001399.s001.tif]

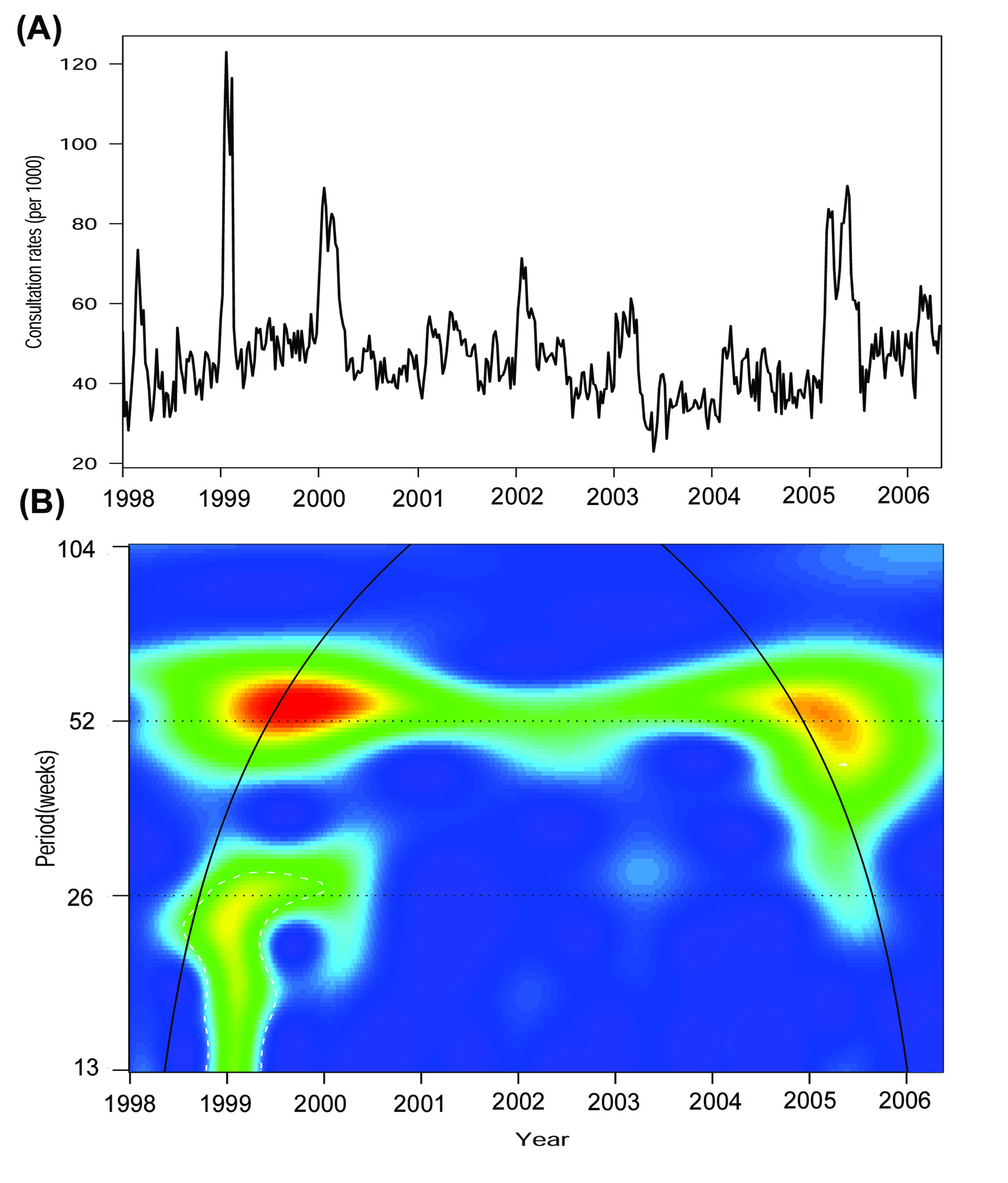

Supplement: Figure S2 — Wavelet analysis for the weekly consultation rates of influenza-like illness in GP. (6.94 MB TIF) [file pone.0001399.s002.tif]

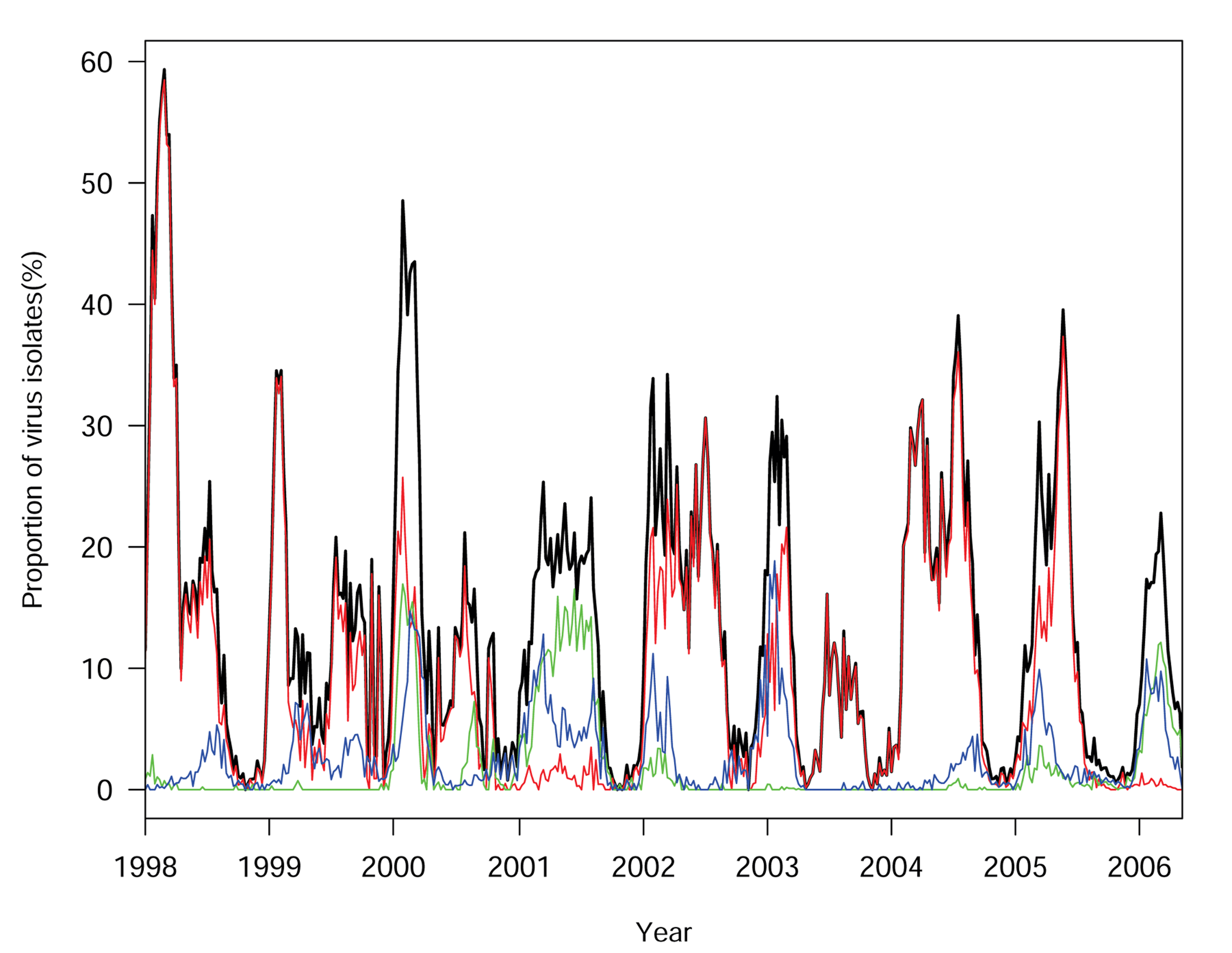

Supplement: Figure S3 — Weekly proportions of isolates positive for influenza subtypes: A(H3N2) (red), A(H1N1) (green), B(blue) and overall proportions (black), 1998-2006. Data were collected from the Department of Health. (3.60 MB TIF) [file pone.0001399.s003.tif]

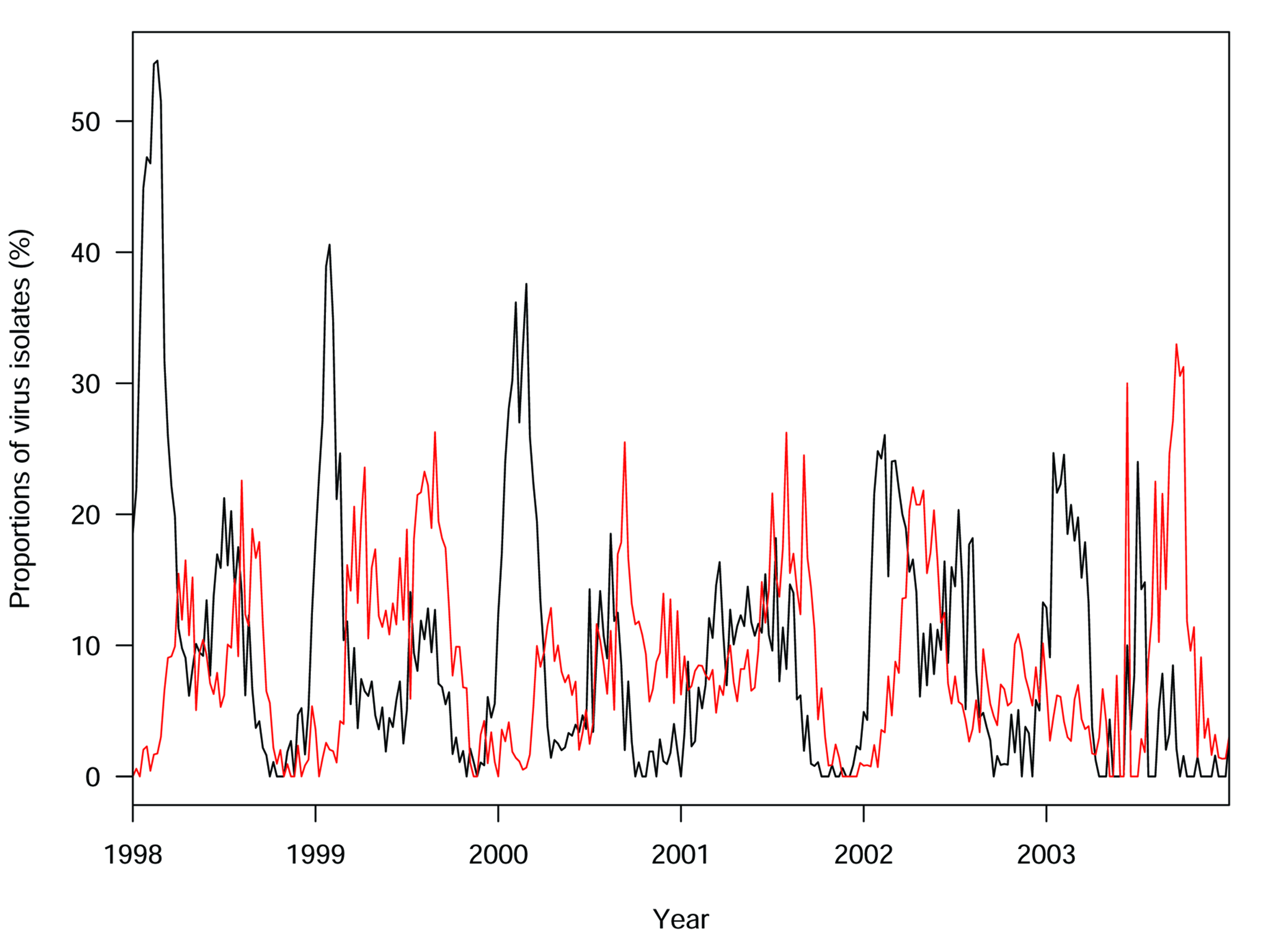

Supplement: Figure S4 — Weekly proportions of isolates positive for influenza (black) and RSV (red), 1998-2003. Data were collected from the Queen Mary Hospital, one of major public hospitals in sentinel surveillance systems of Hong Kong. (3.80 MB TIF) [file pone.0001399.s004.tif]
